# Increased Phase Cone Turnover in 80–250 Hz Bands Occurs in the Epileptogenic Zone During Interictal Periods

**DOI:** 10.3389/fnhum.2020.615744

**Published:** 2020-12-23

**Authors:** Ceon Ramon, Mark D. Holmes

**Affiliations:** ^1^Department of Electrical & Computer Engineering, University of Washington, Seattle, WA, United States; ^2^Regional Epilepsy Center, Harborview Medical Center, University of Washington, Seattle, WA, United States

**Keywords:** EEG ripple, EEG phase cones, epilepsy and phase cones, high-frequency brain signals, cortical phase transitions, phase clusters

## Abstract

We found that phase cone clustering patterns in EEG ripple bands demonstrate an increased turnover rate in epileptogenic zones compared to adjacent regions. We employed 256 channel EEG data collected in four adult subjects with refractory epilepsy. The analysis was performed in the 80–150 and 150–250 Hz ranges. Ictal onsets were documented with intracranial EEG recordings. Interictal scalp recordings, free of epileptiform patterns, of 240-s duration, were selected for analysis for each subject. The data was filtered, and the instantaneous phase was extracted after the Hilbert transformation. Spatiotemporal contour plots of the unwrapped instantaneous phase with 1.0 ms intervals were constructed using a montage layout of the 256 electrode positions. Stable phase cone patterns were selected based on criteria that the sign of spatial gradient did not change for a minimum of three consecutive time samples and the frame velocity was consistent with known propagation velocities of cortical axons. These plots exhibited increased dynamical formation and dissolution of phase cones in the ictal onset zones, compared to surrounding cortical regions, in all four patients. We believe that these findings represent markers of abnormally increased cortical excitability. They are potential tools that may assist in localizing the epileptogenic zone.

## Introduction

Cortical dynamics play an important role to study the behavior of the brain under normal and epileptic conditions. Recently we have shown the oscillatory patterns of phase cone formations near the epileptic spikes (Ramon et al., 2018). Similarly, it is also a possibility that these phase cone patterns might also be higher in epileptogenic areas during interictal periods. This is what we have investigated in this report and found to be true for the high-frequency ripple bands of 80–150 and 150–250 Hz. These findings also complement our earlier results that the phase synchronization index in the low gamma (30–50 Hz) band during interictal periods is higher in the epileptogenic zones.

The phase jumps and related phase cone structures need some introduction. These phase jumps also called phase slips (Pikovsky et al., 2001), arise due to the state transitions of the coordinated activity of cortical neurons in a local neighborhood at mesoscopic (~0.5–1.0 mm) scales and can be utilized to study the dynamical behavior of the cortex under different cognitive conditions (Freeman and Barrie, 2000; Freeman and Rogers, 2002). The electrical activity of the cerebral cortex, in general, is in a dynamic state of criticality (Beggs and Timme, 2012), and the slightest external input, e.g., visual stimuli, or an internal input, e.g., a thought, could induce a state transition which will give rise to a sudden phase jump. This critical behavior of the cerebral cortex is very similar to the triple point of water at the boundary of solid, liquid, and gas phases. Another example will be the sandpile model of self-organized criticality (Bak et al., 1987) in which one keeps adding grains of sand to a sandpile till it collapses. A very simple example will be a pot of boiling water. In the beginning, there will be the formation of small bubbles at a few places at the bottom of the pot which begin to rise toward the top surface of the water. As temperature rises, few more streams of bubbles rise to the surface and one begins to see the spatial formation of a group of bubbles on the surface of the water. This continues till the whole pot of water begins to boil. The spatiotemporal formation of phase cones on the cortical surface is very similar. In the beginning, few isolated phase cones appear on the cortical surface and as more neurons begin to go through phase transitions, there will be the appearance of more phase cones. Some of these phase cones will be isolated and grow in size while others will subside. Also, some phase cones will be stable and will begin to expand slowly in size on the cortical surface (Freeman, 2008). This is a simile of cortical phase cones to the bubbles in a pot of boiling water.

These concepts of criticality have been applied to study the nonlinear brain dynamics (Freeman and Vitiello, 2006, 2010; Freeman, 2008), the cinematic behavior of cortical phase transitions (Kozma and Freeman, 2017), and the behavior of epileptic spikes (Ramon et al., 2018). Thus, phase jumps play an important role to study brain dynamics from the EEG data. These state transitions are also often called phase transitions, cortical phase transitions, or EEG phase transitions in the neuroscience and EEG literature.

These phase transition-related activities show up as small perturbations, i.e., glitches in the EEG data. On the application of Hilbert transform to the EEG data, one gets a sawtooth pattern of the instantaneous phase that can be unwrapped to give an almost linearly increasing phase with respect to time that will have small slow variations. The linear or nonlinear trends in the unwrapped phase can be removed by differencing or taking a derivative (*d/dt*) of the time-series of the unwrapped phase. The phase jump will then show up as a sharp peak or valley whenever there is a slight perturbation in the EEG trace, particularly near the zero crossing line. A peak will represent the growing coordination of firing of neurons in the local neighborhood while a valley will represent the loss of coordination between nearby neurons (Freeman, 2003).

These phase jumps and related spatial phase cones were first observed in the ECoG data (8 × 8 grid, 0.79-mm interelectrode distance) of rabbits (Freeman and Barrie, 2000; Freeman and Rogers, 2002; Freeman, 2003), then in the ECoG data (1 × 1 cm microgrid array with 64 electrodes, 8 × 8 grid, 1.25 mm separation) placed on the right inferior temporal gyrus of a human subject (Freeman et al., 2006a, b), and more recently in 64 channel scalp EEG (Ruiz et al., 2010) and in the high density, 256 channel, scalp EEG data of human subjects (Ramon and Holmes, 2013a, 2015; Ramon et al., 2013, 2018). In spatiotemporal frames, one can see the amplitude and phase-modulated waves in the theta (3–7 Hz) and alpha (7–12 Hz) bands with carrier frequencies in the beta (12–30 Hz) and gamma (30–80 Hz) bands (Freeman, 2004; Myers et al., 2014; Kozma and Freeman, 2017). Aperiodic phase resetting at alpha-theta rates in the scalp EEG of human subjects has also been observed (Freeman et al., 2003). This scalp EEG data was collected with a curvilinear array of 64 electrodes, 3 mm apart, extending 18.9 cm, and placed on the forehead across the midline just below the frontal hairline (Freeman et al., 2003). A common theme in these studies has been to search for spatiotemporal behavior of phase cone structures in the traditional EEG (theta, alpha, beta, and gamma) bands during cognitive tasks, near to the epileptic spikes and during interictal periods. The oscillatory patterns of the phase cone activity near to the epileptic spikes in the low gamma band, 30–50 Hz, and in the ripple band of 80–150 Hz have been examined earlier by us (Ramon et al., 2018). However, no detailed study in the ripple bands (80–150 and 150–250 Hz) during interictal periods has been performed before except reporting of some preliminary results in conference abstracts (Holmes and Ramon, 2014; Holmes et al., 2015). Our newer results are reported here and show that the phase cone activity in the ripple bands during interictal periods is higher as compared to the nearby areas and it could become a complementary noninvasive tool to localize epileptogenic zones from interictal EEG data for presurgical planning purposes.

## Materials and Methods

### Subjects Data

Data sets of four subjects were used for this study. These subjects were all patients at the Harborview Medical Center, the University of Washington for presurgical epilepsy monitoring, and later on, three subjects went through invasive subdural grid and strip electrode recordings. All data sets were collected with the approved Human Subjects Guidelines at the University of Washington. Written permission was obtained from each subject to use these data sets for research purposes. Before invasive recordings, during presurgical evaluations, high-density 256-channel scalp EEG data, also called hdEEG, were collected with an EEG system developed by Electrical Geodesics, Incorporated (EGI), Eugene, OR, USA (now Magstim EGI^1^). For an adult head, from the center of one electrode to the other, the interelectrode separation is approximately 2.0 cm (Tucker, 1993). The data were collected with a sampling rate of 1.0 kHz, i.e., the time difference between two consecutive samples was 1.0 ms. The hdEEG data of each subject during sleep was collected between 2 and 4 h or so. For all four subjects, in the waking state, there was a symmetric 9–10 Hz posterior alpha rhythm that attenuates symmetrically with eye-opening. A central mu rhythm was seen on either side from time to time. Drowsiness was characterized by waxing and waning of the alpha rhythm, slow lateral eye movements, and intermittent generalized theta activity. Stage II sleep was characterized by symmetric V-waves, K-complexes, sleep spindles, and positive occipital sharp transients of sleep. Interictal epileptiform abnormalities (spike and sharp waves) were observed, mainly during drowsiness and stage II sleep. There were no clinical or electrographic seizures. The interictal data for analysis was selected during the waking state and/or during the drowsiness which was free from Stage II sleep-related spindles et cetera.

Interictal invasive subdural ECoG were also collected for three of the four subjects later on with 8 × 8 grid electrodes and strip electrodes to localize the seizure areas. The electrodes on the grid or strips had an exposed surface area defined by 2.3 mm diameter and with center-to-center, inter-electrode separation of 1.0 cm (Johnson et al., 2012). The epileptic sites were localized from seizure activity in ECoG and/or in hdEEG data sets.

Subject #1 was an adult 35 years old female who was a candidate for surgery. The epileptic spikes and seizures were observed over left central-parietal areas, in the vicinity of the somatosensory cortex. Subject #2 was a 20-year-old male with drug-resistant focal epilepsy with complex left partial midline seizures and was a candidate for surgery. Subject #3 had epileptic spikes and seizure activity over midline/anterior frontal regions, with shifting hemispheric preponderance. The activity area was mapped with hdEEG and subdural electrocorticograms (ECoG) recordings. He was a 26-year-old male and was a candidate for surgery. Subject #4 was a 26-year-old female who had seizure activity in left frontal-temporal and inferior midline areas as observed in hdEEG recordings. She declined to go for surgery. All data sets were de-identified and then used for analysis.

### Data Analysis

Interictal scalp hdEEG recordings, free of epileptiform patterns, of 240-s duration, were selected for analysis for each subject. All data sets were at least half an hour away from any epileptiform activity. The data were filtered in broadband of 70–300 Hz with an equiripple filter. The eye-blink and muscle artifacts were removed by use of principal component analysis (PCA) techniques by use of EEGLAB software. The data was then filtered in the appropriate EEG ripple band of 80–150 Hz or 150–250 Hz and Hilbert transform was applied to extract the instantaneous phase and then it was unwrapped which gives almost a linearly increasing time series of the phase.

The equiripple filter was designed based on the Parks-McClellan algorithm (McClellan and Parks, 2005) and was implemented in a MATLAB environment. It is an equiripple linear phase FIR (finite impulse response) filter. The ripples in the passband and the stopbands are of equal height which can be specified while designing the filter. Our bandpass filters were designed with 0 ± 0.25 dB gain in the passband and −60 dB gain (or loss one could say) in the stopband. The transition bands were 1.0 Hz. As an example, the 80–150 Hz passband filter had the following design parameters: sampling frequency, *Fs* = 1 kHz, *F*_stop1_ = 79 Hz, *F*_pass1_ = 80 Hz, *F*_pass2_ = 150 Hz, *F*_stop2_ = 151 Hz, passband gain = 0.0 dB, ripple = 0.5 dB, stopband gain = −60 dB. The filter order was 2201. Similarly, other filters were designed by use of the Filter Designer toolbox in MATLAB. The EEG data were filtered with the “filtfilt” function in MATLAB which does not introduce any phase distortions in the filtered data by filtering in both the forward and reverse directions.

The Hilbert transform is a very common technique to extract the analytic amplitude and phase from the EEG data (Barlow, 1993; Freeman et al., 2003; Freeman, 2007; Myers and Padmanabha, 2017; Mortezapouraghdam et al., 2018). With the Hilbert transform, a ±π phase shift is introduced at each sinusoidal peaks/valleys and also wherever there is a slight perturbation in the EEG trace. The instantaneous phase, also often called the analytic phase in the EEG literature, comes out as a sawtooth pattern after taking the Hilbert to transform which after unwrapping gives a linearly increasing phase with slight glitches corresponding to the perturbations in the EEG trace. The unwrapping was done by use of the “unwrap” command in MATLAB. It adds 2π at every reset point in the sawtooth pattern of the instantaneous phase (Mortezapouraghdam et al., 2018). More details about the algorithm and the MATLAB script are described in the documentation for the “Unwrap” in the DSP Systems Toolbox, and are also described in the literature (Karam and Oppenheim, 2007).

**Figure 1:**
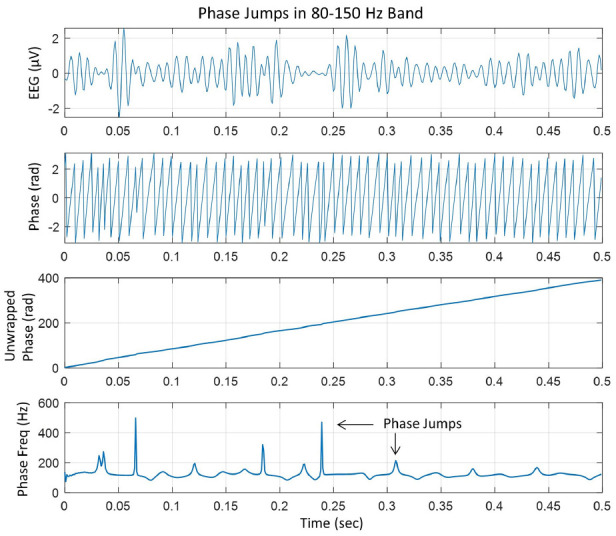
(Top) EEG trace filtered in the 80–150 Hz ripple band, (second plot from the top) phase in radians extracted after taking Hilbert transform of the EEG trace, (third plot from the top) unwrapped phase in radians which is almost linear with slight variations, and (bottom plot), taking the derivative of the unwrapped phase and dividing by 2π to get the instantaneous phase-frequency in cycles/s or Hz. The peaks are phase jumps that appear whenever there is a slight perturbation, particularly near the zero crossing line in the EEG trace.

In our analysis, the linear trend in the unwrapped phase was removed by taking the first-order derivative (*d/dt*) of the unwrapped phase. From this, the instantaneous phase-frequency was computed. An example of these steps is given in Figure 1.

The top plot shows the EEG in 80–150 Hz band at one of the electrodes in the left central midline area. It has high-frequency spindles which are very common (Mooij et al., 2017; Boran et al., 2019, 2020). The second plot from the top is the instantaneous phase after taking the Hilbert transform of the EEG data and the third plot from the top is the unwrapped phase. The derivative of the unwrapped phase was converted into the instantaneous frequency in cycles/s (or Hz) by dividing by 2π, which is given in the bottom plot which has several peaks related to the cortical phase transitions. Some peaks are sharp which will form transient spatiotemporal phase cones and will disappear quickly. Some peaks are slightly wider as compared with sharp peaks and will tend to form stable spatiotemporal phase cones which could last for several digitization points, e.g., 2–5 ms, for the data collected at 1 kHz. In general, it is very common to look for stable phase cone patterns for three consecutive digitization steps (Ramon and Holmes, 2013a; Ramon et al., 2013, 2018; Ruiz et al., 2010).

These phase jumps were extracted for all 256 channels over the 240-s long data set. Out of which stable phase cone structures were extracted with ms resolution. These procedures have been described earlier by us (Ramon et al., 2018) and also by others (Ruiz et al., 2010; Freeman, 2015). A summary is given here.

**Figure 2:**
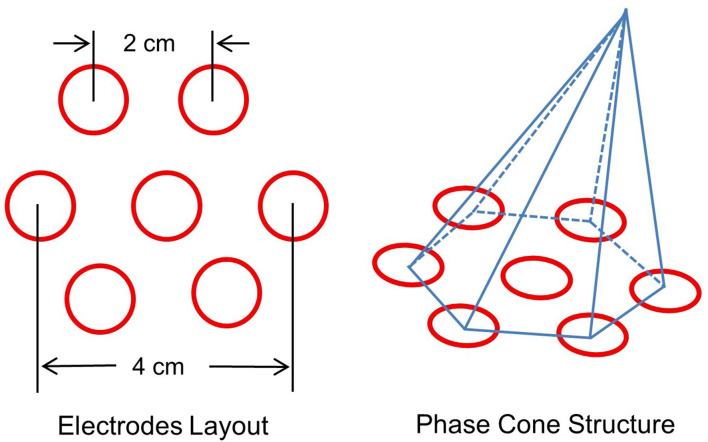
(Left) Layout of a center electrode and six nearby electrodes, and (right) a schematic representation of the phase cone which arises due to the coordinated activity of cortical neurons at mesoscopic scales. The interelectrode separation is 2.0 cm.

Several criteria were applied to select stable phase cone patterns. These included: (1) phase-frequency was within the temporal band, e.g., 80–150 Hz or 150–250 Hz; (2) sign of spatial gradient and maximum or the minimum did not change for at least three consecutive time samples; and (3) the frame velocity should be within the range of conduction velocities of cortical axons, 1–10 m/s (Swadlow, 1994; Swadlow and Waxman, 2012; Ramon et al., 2018). The rate of change in phase with distance (rad/mm) was computed from the spatial location of the electrodes on the scalp. An idealized layout of seven electrodes on the scalp surface is given in Figure 2. It shows one central electrode and six nearby electrodes. The interelectrode separation is about 2 cm which gives about 4 cm separation between diametrically opposite electrodes. An idealized representation of a phase cone on a group of six electrodes is also shown. The diameter of the phase cone was approximately equal to 4 cm or less. This choice was made to look for the local formation of stable phase cones which do not expand to the whole cortical surface of about 20 cm diameter for an adult head. This gives a finer spatial resolution to localize the epileptogenic sites.

Based on these criteria, stable clusters of frames in each second long EEG data were computed and an averaged rate of phase cone clusters over 240 s was computed. Color intensity plots of an averaged rate of phase clusters were constructed using a montage of the layout of 256 electrode positions displayed as if one was looking on top of the subject’s head. In all the spatial plots in the “Results” section, the nose is on the top, the back of the neck is at the bottom, the left of the subject is on the left side of the plot and the right side of the subject is on the right side of the plot.

### Surrogate Data Analysis

Surrogate data testing was performed to make sure that our results are real and are not due to chance occurrences. Procedures for performing this analysis are described elsewhere (Prichard, 1994; Kugiumtzis, 2002; Lancaster et al., 2018). The main points are that the signal processing steps, including filtering differentiation, etc., should be kept the same for the real and the surrogate data. Otherwise, comparative analysis between the real and the surrogate data could be biased. The most simple procedure is to generate random data from a subset of real data and perform the same analysis on both data sets. If the results are significantly different then the phenomena or process under investigation is genuine and not a chance occurrence. For our work, the surrogate data was generated by randomly shuffling the real EEG data. A 240 s long filtered 256 channel EEG data, as described above, from one of the subjects was converted into a vector, randomly shuffled using the “randperm” command in MATLAB, and then rearranged as 256 channel surrogate EEG data. This data was then used to compute the rate of formation of stable phase cones (counts/s) over 240 s interictal period. The maximum rate of the formation of stable phase cones on all electrodes was extracted. This process was repeated for 100 trials. The averaged value of the maximum rate of stable phase cones over 100 trials was: 2.31 ± 0.98 (*n* = 100) counts/s for the ripple band of 80–150 Hz. This process was repeated for the fast ripple band of 150–250 Hz. The averaged value of the maximum rate of stable phase cones over 100 trials was: 12.12 ± 3.15 (*n* = 100) counts/s. These values (< = 12.12 counts/s) for the randomly shuffled EEG data are much lower as compared with the actual EEG data (50–310 counts/s) shown in the figures in the “Results” section. Thus, our results are genuine, above the random noise, and are due to neurophysiological processes in the brain.

## Results

**Figure 3:**
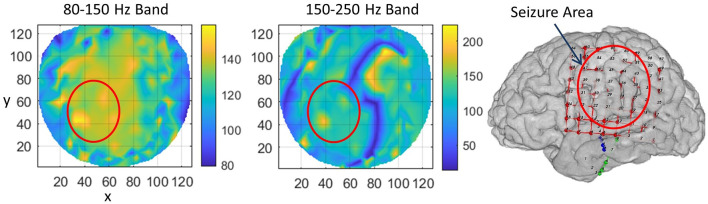
The rate of formation (counts/s) of stable phase cone clusters averaged over a 240-s interictal period for subject #1 in the 80–150 Hz and 150–250 Hz bands are given in the left and middle plots, respectively. The color bar indicates the rate in counts/s. (Right plot) The grid layout for electrocorticograms (ECoG) data collection with red ellipse showing the area where seizures and epileptogenic activities were observed.

For subject #1, the stable phase cone clusters are given in Figure 3. The left two plots are for the stable phase cone patterns for the 80–150 and 150–250 Hz bands, respectively. The right plot is the cortical surface with subdural grid and strip electrodes for ECoG recordings. The red ellipse in the left central-parietal areas is the main area where the majority of the seizures and epileptogenic activities, such as spikes were observed intermittently for 5 days. The hot spots of the stable phase cone clusters in the 80–150 Hz band (left plot in Figure 3) are enclosed in the red circle depicting the possible seizure area. The maximum value of 158 counts/s is at the *x, y* coordinate location of (34, 42). The horizontal (*x*) and vertical (*y*) axes are in arbitrary length units based on the normalized location of 256 electrode positions on a flat surface. The stable phase cone activity is spread in a wide area. This possibly could be related to the epileptogenic cortical network activity during interictal periods which possibly could be spread on an interconnected wide area cortical networks. However, the hot spots of maximum activity are in the epileptogenic zone which was identified from ECoG recordings. Compared to this, the stable phase cone clustering activity in the fast ripple band of 150–250 Hz is confined to a single spot (183 counts/s) approximately at the same coordinate location of (37, 42). Refer to the middle plot. This plot does not show a widespread network activity as seen in the 80–150 Hz band (left plot). There is another bright lengthy spot in the right front central (*x* = 70–100, *y* = 70–105) area. This possibly could also be due to the large area cortical networks involved in the cortical phase transitions. This is explored in detail in the “Discussion” section.

**Figure 4:**
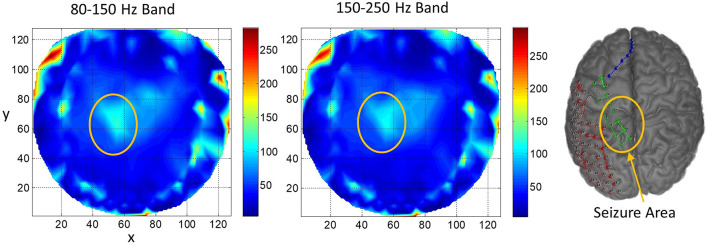
The rate of formation (counts/s) of stable phase cone clusters averaged over a 240-s interictal period for the subject #2 in the 80–150 and 150–250 Hz bands are given in the left and middle plots, respectively. The color bar indicates the rate in counts/s. (Right plot) The grid and strip electrodes layout for ECoG data collection. The yellow-orange ellipse marks the area where seizures and epileptogenic activities were observed.

For subject #2, the stable phase cone cluster activity is given in Figure 4. During ECoG recordings, the seizure activity was observed in the complex left partial midline areas. It is marked with a yellow-orange ellipse. For both bands, the rate of formation (counts/s) of stable phase cone clusters is higher in the epileptogenic area as compared with nearby areas. The peak rate is about 115 counts/s for the 80–150 Hz band and about 107 counts/s for the 150–250 Hz band. These are averaged values over 240 s. There is diffused phase cone activity outside the elliptical area, particularly in the north-east direction spread over *x* = 60–85 and *y* = 55–90. This will correspond to the right midline central area of the brain. Most probably, this activity is related to the cortical networks in the vicinity of the main focus of the epileptogenic area marked with an ellipse. There are bright spots (~270 counts/s) in the left front temporal area and also in the right front temporal and right central temporal areas. These probably are due to muscle artifacts which can be suppressed by the use of image processing techniques. However, we decided to include it here in these plots to show that phase cone formations are strong for muscle artifacts. Overall, the rate of formation of stable phase cone clusters is also strong in the epileptogenic areas.

**Figure 5:**
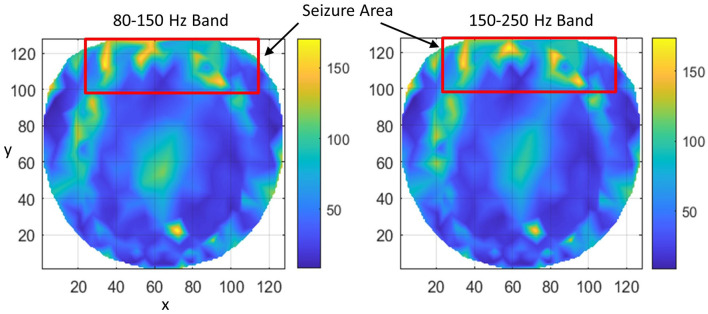
The rate of formation (counts/s) of stable phase cone clusters averaged over a 240-s interictal period for subject #3 in the 80–150 and 150–250 Hz bands are given in the left and right plots, respectively. The color bar indicates the rate in counts/s. The seizure activity area is marked with a red rectangle which shows several hot spots.

Subject #3 had epileptic spikes and seizure activity over midline/anterior frontal regions, with shifting hemispheric preponderance. This region is marked with a rectangle. Refer to Figure 5. There are several hot spots in both bands in the rectangular area which signify the higher rate of formation of phase cone clusters as compared with nearby areas and most probably are related to the silent interictal epileptogenic activity. There are some scattered bright spots in the left central area which possibly could be the continuation of the left frontal epileptogenic activity. There is a bright spot around the coordinate location of (74, 23) which could be due to some other activity in the brain and not related to epileptogenic activity in the frontal regions. It is a possibility that it is a separate epileptogenic activity which was not observed in the hdEEG recordings but showed up in the interictal phase cone analysis. Sometimes it does happen that the stochastic analysis of EEG power does not show the exact and complete spatial patterns of epileptogenic activity while they show up in more detail with the stochastic analysis of EEG phase synchronization (Ramon et al., 2008). The diffused midline central activity area, possibly, could be an epileptogenic activity or unidentified background brain activity during the Stage I sleep. However, this needs to be confirmed with additional studies.

**Figure 6:**
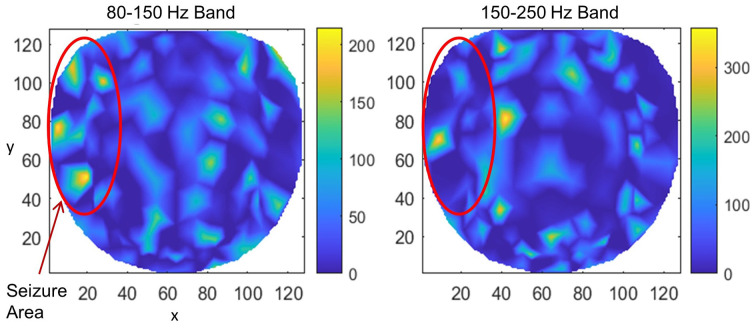
The rate of formation (counts/s) of stable phase cone clusters averaged over a 240-s interictal period for subject #4 in the 80–150 and 150–250 Hz bands are given in the left and right plots, respectively. The color bar indicates the rate in counts/s. The epileptic spikes and seizure activity area is marked with a red ellipse. The left plot shows several hot spots in the left temporal frontal, central and parietal areas. The activity in the 150–250 Hz band, the right plot, has one hot spot in the left temporal central area and another hot spot at the right side of the red circle which could also be related to the silent interictal epileptic activity.

Results for subject #4 are given in Figure 6. This subject had epileptic spikes and seizure activity in the left frontal-temporal and inferior midline areas. In the left plot for 80–150 Hz band, there are three distinct hot spots where the rate of formation of stable phone cones is very high (150 −210 counts/s) as compared with nearby areas where the rate is low (0–50 counts/s). This activity is mainly confined to the left frontal, central, and parietal temporal areas. The cortical activity in the inferior midline area could get picked up by the scalp electrodes in the left temporal areas which possibly could be the additional sources of the formation of the observed hot spots in the left temporal areas. The activity in the 150–250 Hz band is limited only to one hot spot in the left temporal central with a peak rate of 307 counts/s averaged over a 240-s interictal period. There is another hot spot at the right side of the red circle at the *x, y* coordinate location of (40, 80). Its peak rate is 342 counts/s and is also probably related to the silent epileptic network activity during the interictal period. There are other numerous visible spots in the range of 100–135 counts/s for the 80–150 Hz band and between 180–250 counts/s for the 150–250 Hz band. These, most probably, are related to the spontaneous background activity of the brain and not related to any epileptogenic activity.

The peak values in the epileptogenic zone were compared with nearby baseline values for all four subjects. These values are given in Table 1. The peak values are significantly higher in the epileptogenic zones. This was determined by the paired *t*-test between the values (counts/s) for the background and epilepsy, i.e., epileptogenic zone.

**Table 1:** Change in peak value of the phase cone rate (counts/sec) in the epileptogenic zone as compared with the nearby background rate.

80–150 Hz				150–250 Hz		
	Background	Epilepsy	% Change	Background	Epilepsy	% Change
Subject #1	100	158	58%	72	183	154%
Subject #2	45	115	155%	47	107	128%
Subject #3	37	160	332%	34	162	376%
Subject#4	25	210	740%	50	307	514%
Mean % change (all subjects)			321 ± 301			293 ± 185
Statistical Significance		*p* < 0.03		*p* < 0.045		

## Discussions

The overall summary of our results is that the rate of formation of stable phase cone cluster patterns during interictal periods is higher in the epileptogenic zones in the 80–150 and 150–250 Hz bands and are above the value of 12.12 counts/s for the surrogate data. This we have shown in four subjects. These are encouraging preliminary findings and need to be confirmed with a larger subject population. These findings suggest that there is a silent background epileptic network activity in the high frequency (80–250 Hz) EEG band during interictal periods which possibly could be a physiological marker to localize and isolate epileptogenic zones. Such information derived during interictal periods could become a useful tool for brain electrical stimulation to control the onset of epileptic seizures.

The observed phase cone activity in both high frequency (80–150 and 150–250 Hz) bands is often spread outside of the mapped epileptogenic zones. This could be due to the large area cortical networks involved in the cortical phase transitions that give rise to the spatiotemporal formation of phase cones (Freeman and Vitiello, 2006; Freeman, 2008). The phase cone activity related to the background cognitive processes in the brain is also observed along with the epileptogenic activity in the spatial plots. We have not seen any studies which quantify the phase cone formations due to spontaneous brain activity in healthy subjects or subjects with epilepsy. Thus, it is difficult to quantify and separate the epileptic activity from the background brain activity. This has to be a topic for a carefully designed future study. However, in general, the rate of phase cone formations related to the background brain activity during the Stage I sleep will be much lower than the epileptogenic activity. This is what we are observing in all four subjects. These observations are listed in Table 1. In summary, the observed hot spots in the epileptogenic zones are definitely related to the silent epileptic activity during interictal periods which are above the spontaneous brain activity during the stage I sleep.

The muscle artifacts are a problem, particularly on the facial and back of the neck electrodes. The frequency spectra of the electromyographic (EMG) signal and the EEG are very similar (Whitham et al., 2011; Muthukumaraswamy, 2013) which makes it difficult to use signal processing techniques to separate the muscle activity from the brain activity in the scalp EEG data. The independent component analysis (ICA) or PCA are two of the best tools to remove these artifacts from the EEG data. In some subjects, the electrodes on the face, forehead, and back of the neck, below the skull, pick up a lot of muscle activity along with the basal brain activity. However, even with PCA or ICA analysis, it is difficult to get rid of all the muscle activity in these electrodes. Often in the component spectra of PCA or ICA, one can see the residual muscle activity along with a strong component of the brain activity. In spatial component maps, the focus of the activity is generally observed in the vicinity of the skin, skull bone, and the neocortex, and it is difficult to separate the dipoles related to the muscle artifact from the brain activity. So, one has to make a judicious choice to keep some of the ICA or PCA components that have mixed brain and muscle activity. These electrodes will show the phase cone activity due to muscle activity and also due to brain activity. Only in the data of subject #2, we had strong muscle activity on outer rim electrodes (face, near to ears, and the neck). This subject had complex left partial midline seizures so the muscle activity at the outer rim electrodes was not a confounding factor. For the other three subjects, we did not have this problem.

Our work reported here complements our previous work on the stable phase cone formations during interictal periods in the theta (3–7 Hz), alpha (7–12 Hz), beta (12–30 Hz), and low gamma (30–50 Hz) bands (Ramon et al., 2008; Ramon and Holmes, 2013a). It also complements the oscillatory patterns of phase cone formations near epileptic spikes (Ramon et al., 2018). These combined studies show that the rate of formation of stable phase cones is higher in the vicinity of epileptogenic zones in different low and high-frequency EEG bands. Also, we have shown earlier that the stochastic behavior of phase synchronization index exhibits a complex behavior, and the phase-amplitude cross-frequency couplings are reduced and spatial patterns are fragmented during interictal periods in the epileptogenic zones (Ramon and Holmes, 2012, 2013a, b). These studies were performed in the lower (3–50 Hz) EEG frequency bands. The stochastic behavior of the phase synchronization index was higher in the beta (12–30 Hz) and low gamma (30–50 Hz) bands but depressed in the theta (3–7 Hz) and alpha (7–12 Hz) bands. These earlier findings of the stochastic behavior of the phase synchronization index are also of interest and should be extended to examine this behavior in the high frequency (80–250 Hz) EEG bands to localize the epileptogenic sites in the brain in a noninvasive fashion. In summary, we believe that the findings described in this article, namely, that focal area of increased phase cone turnover are markers of abnormal cortical excitability, and as such, may eventually be utilized as tools to assist in the noninvasive localization of epileptogenic cortices.

## Data Availability Statement

All datasets used in this study are available from the authors without undue reservation. In addition, in the near future we will submit our datasets to some repository, such as, GitHub.

## Ethics Statement

The studies involving human participants were reviewed and approved by Human Subjects Division, University of Washington, Seattle, WA 98195, USA. The patients/participants provided their written informed consent to participate in this study.

## Author Contributions

Data was collected by MH. Data analysis was done by CR and MH. Discussions of results and writing was done jointly by both authors. All authors contributed to the article and approved the submitted version.

## Conflict of Interest

The authors declare that the research was conducted in the absence of any commercial or financial relationships that could be construed as a potential conflict of interest.

## Footnotes

^1^www.egi.com
